# Supplementary material for: Designing optimal COVID-19 testing stations locally: A discrete event simulation model applied on a university campus
Source: PLoS One. 2021 Jun 29;16(6):e0253869. doi: 10.1371/journal.pone.0253869 (PMC8241042; doi:10.1371/journal.pone.0253869)
Supplement: S1 Appendix — (DOCX) [file pone.0253869.s002.docx]

S1 Appendix. Gantt diagrams of the testing process

In the case where all tasks performed in serial with only one operator and one single machine of each kind available for heating, transferring, preparing/pre-testing, and testing available on-site (see S1 Fig), it has been found that one batch (i.e., 384 samples/vials) can be tested in 11.5 hours, which is far from the objective given of 10,000 samples tested per day.


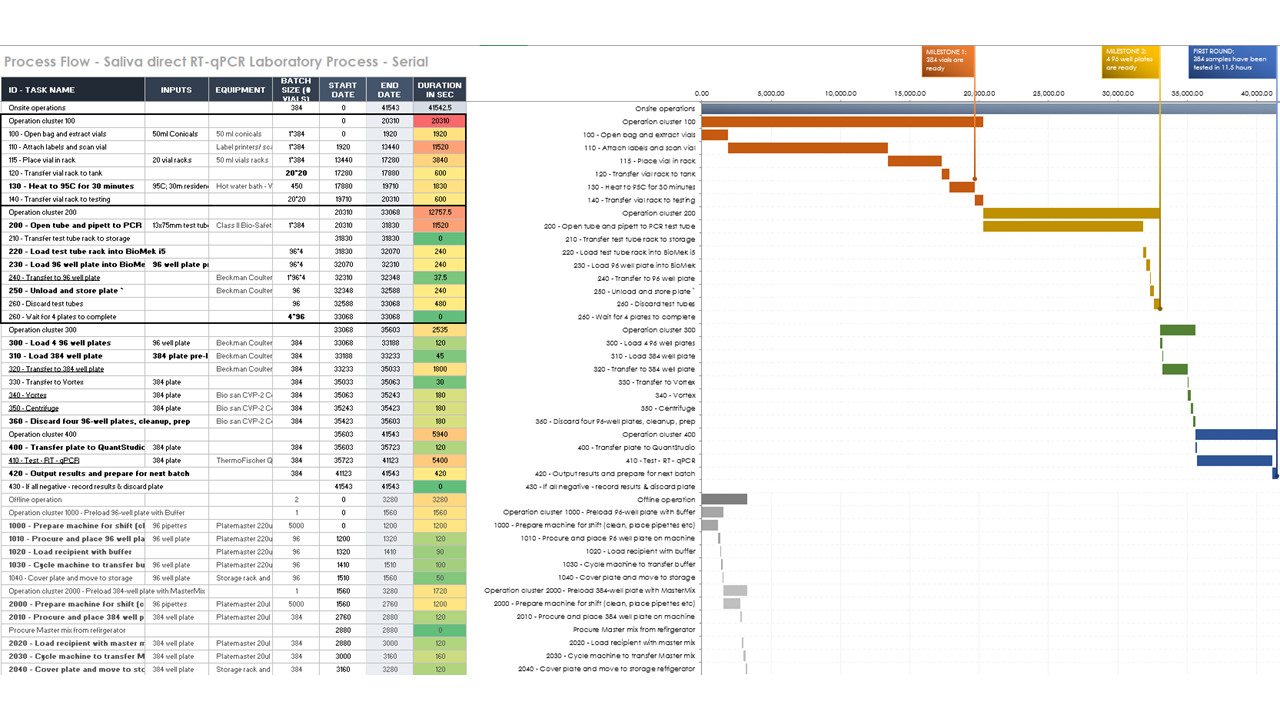


**S1 Fig. Gantt diagram of the testing process (all tasks in serial)**

In the Gantt chart of S2 Fig, by increasing the number of operators (from one to ten) for the most time-consuming tasks (e.g., ID tasks 100-140), and by doubling the number of testing equipment (from one to two), it would take 3.8 hours for testing the first batch of the day. For the second and upcoming batch, with the same setup, several tasks could be parallelized, e.g., the ID task 130, saving 0.5 hour) and having a second testing machine on-site could save up another 0.75 hour in this configuration, as the ID task 410 might become a bottleneck with an accumulation (a queue in DES language) of samples to be tested.


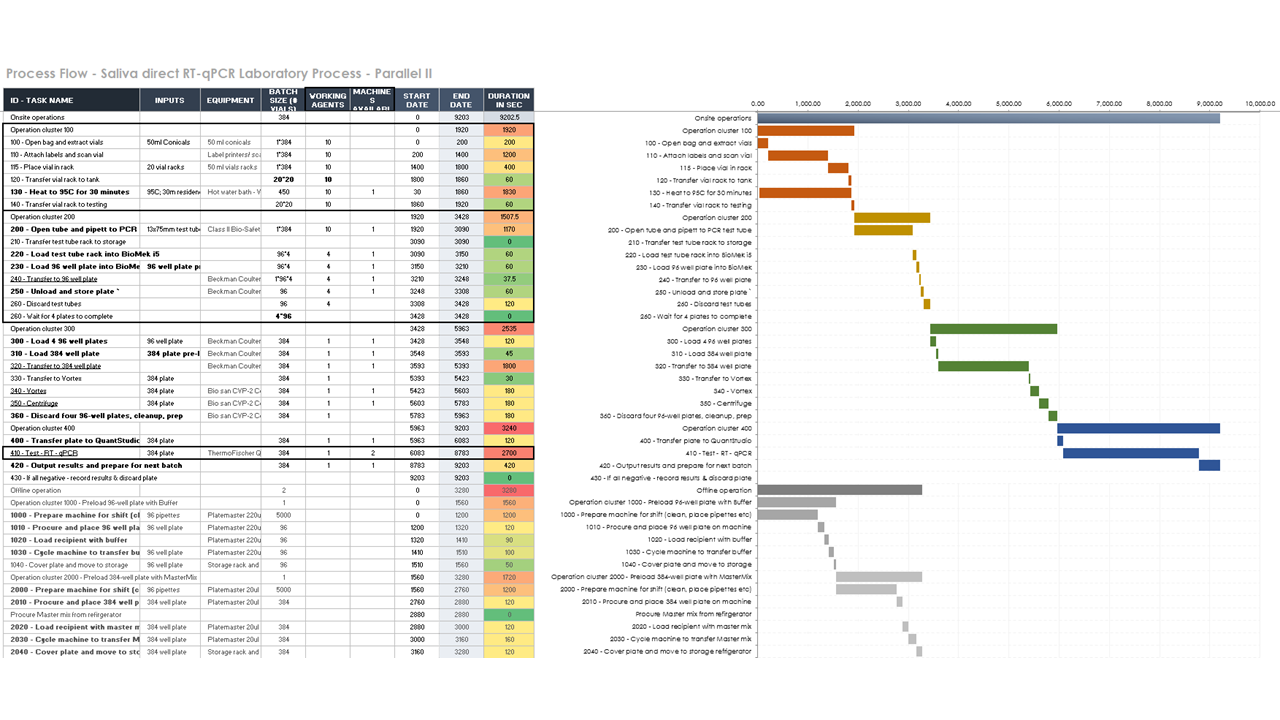


**S2 Fig. Gantt diagram of the testing process (with several key tasks parallelized)**
