# Supplementary material for: Designing optimal COVID-19 testing stations locally: A discrete event simulation model applied on a university campus
Source: PLoS One. 2021 Jun 29;16(6):e0253869. doi: 10.1371/journal.pone.0253869 (PMC8241042; doi:10.1371/journal.pone.0253869)
Supplement: Attachment — Submitted filename: Response to Reviewers.docx [file pone.0253869.s003.docx]

**Response to Reviewers – Manuscript PONE-D-21-16234 – Major revisions**

Dear Academic Editor and Reviewers,

Thank you for giving us the opportunity to revise the manuscript PONE-D-21-16234 entitled “Designing optimal COVID-19 testing stations locally: a discrete event simulation model applied on a university campus”. We are pleased to re-submit for publication this revised article before the due date, July 18, 2021. We would like first to thank the Editor and the two Reviewers for their highly constructive criticisms in improving this paper. Indeed, the manuscript has certainly benefited from their insightful comments and meaningful suggestions, notably to enhance its scientific soundness and completeness.

We look forward to hearing from you regarding our submission and to respond to any further questions or comments you may have,

Thank you again for your time and consideration,

Sincerely, The authors

**Academic Editor:** Thippa Reddy Gadekallu

Thank you for submitting your manuscript to PLOS ONE. After careful consideration, we feel that it has merit but does not fully meet PLOS ONE’s publication criteria as it currently stands. Therefore, we invite you to submit a revised version of the manuscript that addresses the points raised during the review process. Based on the comments received from the reviewers and my own observation, I recommend major revisions for the article. Please submit your revised manuscript by Jul 18 2021 11:59PM.

🡪 Thank you for considering our research work to be published in PLOS ONE. We hope that the present responses and additional explanations, as well as the changes and add-ons made in the manuscript accordingly, address adequately the remarks and suggestions made by Reviewers #1 and #2.

Please include the following items when submitting your revised manuscript:

- A rebuttal letter that responds to each point raised by the academic editor and reviewers. You should upload this letter as a separate file labeled 'Response to Reviewers'.
- A marked-up copy of your manuscript that highlights changes made to the original version. You should upload this as a separate file labeled 'Revised Manuscript with Track Changes'.
- An unmarked version of your revised paper without tracked changes. You should upload this as a separate file labeled 'Manuscript'.

🡪 The present rebuttal letter includes specific responses - point-by-point - in blue font to each reviewer’s comments and suggestions. You will also find two versions of the revised manuscript: one with changes visible highlighted (showing clearly the changes made in response to the reviewers’ comments, as required), and one unmarked version.

Journal Requirements:

When submitting your revision, we need you to address these additional requirements.

1. Please ensure that your manuscript meets PLOS ONE's style requirements, including those for file naming. The PLOS ONE style templates can be found at

https://journals.plos.org/plosone/s/file?id=wjVg/PLOSOne_formatting_sample_main_body.pdf and

https://journals.plos.org/plosone/s/file?id=ba62/PLOSOne_formatting_sample_title_authors_affiliations.pdf

🡪 The style of our manuscript has been revised accordingly.

2.Please amend either the title on the online submission form (via Edit Submission) or the title in the manuscript so that they are identical.

🡪 This has been amended.

3.Thank you for stating the following in the Acknowledgments Section of your manuscript:

"This material is partially based upon the initial work and process flow provided by Christian Messmacher, from the SHIELD team. Any opinions, findings, and conclusions, or recommendations expressed in this publication are those of the authors and do not necessarily reflect the views of the University of Illinois or the SHIELD Team."

We note that you have provided funding information that is not currently declared in your Funding Statement. However, funding information should not appear in the Acknowledgments section or other areas of your manuscript. We will only publish funding information present in the Funding Statement section of the online submission form.

Please remove any funding-related text from the manuscript and let us know how you would like to update your Funding Statement. Currently, your Funding Statement reads as follows:

"The authors received no specific funding for this work."

🡪 This is actually correct. We have not received any specific funding for this work, just some initial data as mentioned in the Acknowledgments Section. This is now mentioned more clearly in the Acknowledgments Section to avoid any misunderstanding.

Additionally, because some of your funding information pertains to commercial funding, we ask you to provide an updated Competing Interests statement, declaring all sources of commercial funding.

🡪 As we have not received any specific funding for this work, and as we are not aware of any competing interest, we believe this does not apply to our case.

**Reviewer #1:**

| Comments of Reviewer #1 | Point-by-point answers |
| --- | --- |
| 1. The authors are advised to check the error in the title (By mistake 0 is added in the title). | This has been checked and corrected. |
| 2. Secondly, overall, the language of the paper and grammatical error needs to be fixed throughout the paper, At present it is not up to the mark of the journal's expectations. | Thank you for pointing that out. The entire manuscript has been carefully proofread. See “Revised Manuscript with Track Changes”. |
| 3. The font size and font style of the research paper needs to be according to the template instructions | This point has also been mentioned by the Academic Editor. We made all the necessary changes meet PLOS ONE’s style requirements. |
| 4. Related work and literature review is very weak in the paper, authors are advised to be detailed in this section. | The “Related work” section has been reworked and significantly augmented using the references provided by the two reviewers, combined with additional state-of-the-art papers. |
| 5. Authors have mentioned Fig 2. Overview of the modeling approach, but there is not figure 2 in the paper, | Fig 2 is available in the paper and introduced in the second paragraph of the “Materials and methods” section. |
| Lastly, authors are advised to format the references section and add below references:  a) Srivastava D., Kohli R. and Gupta S. 2017 Advances in Computer and Computational Sciences (Springer) Implementation and statistical comparison of different edge detection techniques 211-228  b) Gomathi, S.; Kohli, R.; Soni, M.; Dhiman, G.; Nair, R. Pattern analysis: Predicting COVID-19 pandemic in India using AutoML. World J. Eng. 2020 | Thank you for sharing these two relevant studies. While the first one (a) seems out of scope for our present manuscript, the second one (b) has been integrated in the manuscript.  The references are now formatted in accordance with Journal’s style requirements. |

**Reviewer #2:**

| Comments of Reviewer #2 | Point-by-point answers |
| --- | --- |
| • Introduction section can be extended to add the issues in the context of the existing work | Thank you for this suggestion. Introduction section has actually been augmented (see “Related work” section). |
| • Literature review techniques have to be strengthened by including the issues in the current system and how the author proposes to overcome the same. | The “Related work” section provides both inspiration sources to develop the proposed model, including contributions of existing studies/papers, as well as their limitations and gaps/lacks, justifying as such the relevance of our research work. In all, note eight new papers, including the ones suggested by Reviewers #1 and #2 have been added to strengthen the manuscript. |
| • There are some grammatical and editing problems in English. English presentation should be further polished | As mentioned to Reviewer #1, the whole manuscript has been carefully proofread. |
| • The objective of the research should be clearly defined in the last paragraph of the introduction section. | The objective of the study are defined in the last paragraph of the Introduction section, as suggested. |
| • Add the advantages of the proposed system in one quoted line for justifying the proposed approach in the Introduction section. The authors can add the few advantages of deep learning for COVID -19 diagnosis. The following papers can be referred. Deep learning and medical image processing for coronavirus (COVID-19) pandemic: A survey.  Authors can refer An Incentive Based Approach for COVID-19 planning using Blockchain Technology. | The advantages (relevance and utility) of the proposed DES model are now clearly stated at the end of the Introduction section.  Thank you for sharing these valuable studies. It has been included in the conclusion section as promising perspective for future work, e.g. combining such machine learning approaches (see also reference (b) suggested by Reviewer #1) for COVID-19 diagnosis/prediction with engineering simulation tools. |
